# Applying Community-based System Dynamics to promote child health equity: the case of healthy and fit kids in Milwaukee, WI

**DOI:** 10.3389/fpubh.2024.1375284

**Published:** 2024-11-14

**Authors:** Yvonne D. Greer, Debra Nevels, Amy Meinen, Amy L. Korth, Travis R. Moore, Julia Appel, Kelsey Werner, Larissa Calancie, Andre Lee Ellis, Niky Espy, Shantel Hendricks, Tanya Johnson, Vanessa D. Johnson, Danielle Nabak, Viola Rembert, Christopher Simenz, Nicole Weeks, Angelia Wilks-Tate, Christina D. Economos

**Affiliations:** ^1^Y-EAT Right, Nutritional Consultant for Healthy Living, Milwaukee, WI, United States; ^2^Milwaukee County Organizations Promoting Prevention (MCOPP) Healthy & Fit Kids Strategic Planning Committee, Milwaukee, WI, United States; ^3^Medical College of Wisconsin Cancer Center, Medical College of Wisconsin, Madison, WI, United States; ^4^HealthTIDE, School of Human Ecology, University of Wisconsin, Madison, WI, United States; ^5^ChildObesity180, Friedman School of Nutrition Science and Policy, Tufts University, Boston, MA, United States; ^6^School of Social Work, Boston College of Social Work, Boston, MA, United States

**Keywords:** systems thinking, Community-based System Dynamics, community coalition, child health, health equity, transformative learning, causal-loop diagram, policy

## Abstract

**Background:**

Child health equity is influenced by complex systemic factors, including structural racism, socioeconomic disparities, and access to resources. Traditional public health interventions often target individual behaviors, but there is a growing need for systems approaches that address these root causes. This study examines coalition members’ perspectives on promoting child health equity in Milwaukee as a result of participating in an intervention that includes Community-based System Dynamics (CBSD).

**Methods:**

In this case study, we used a mixed-methods approach to describe 10 coalition members’ perspective shifts over 6 months, after participating in CBSD activities. These activities generated a causal-loop diagram to map the systemic factors influencing child health. Data collection included pre-post interviews and surveys. The data analysis involved thematic analysis of the qualitative data from interviews, which was then integrated with the open-ended survey responses. The themes identified were cross-referenced with the causal-loop diagram factors to validate and refine the understanding of systemic influences on child health.

**Results:**

Post-intervention, coalition members shifted their focus from individual health behaviors to systemic drivers, particularly structural racism and socioeconomic disparities. The causal-loop diagram helped identify leverage points and fostered a readiness for local collective action and policy advocacy.

**Conclusion:**

Integrating CBSD into public health coalitions can shift focus from individual behaviors to systemic causes, enabling more effective interventions. This approach offers valuable insights for promoting child health equity through holistic, community-driven strategies and public policy reforms.

## Introduction

The focus on promoting healthy child weight for optimal growth and development has evolved to emphasize the critical role of social and structural determinants of health in shaping long-term child health outcomes (1). This paradigm shift is a response to the growing recognition of the profound impact that social and structural factors have on the well-being of children (2–4). Within this context, coalitions, comprising various multi-sector agencies and community groups, have actively explored the value of prioritizing equity, community engagement, and restorative justice practices to gain a deeper understanding of the conditions that influence optimal child development and well-being (5–7). In this case study, we aim to document the changes in coalition members’ perspectives on promoting child health equity in Milwaukee as a result of participating in a Stakeholder-driven Community Diffusion theory-informed intervention, which includes the application of Community-based System Dynamics (8, 9). We draw connections between changes in coalition members’ perspective shifts and changes in the Coalition’s future work. We also link these changes to existing literature on coalitions, child healthy weight, and asset-based community development, aiming to inform the efforts of child health promotion coalitions and practitioners (10, 11). Additionally, our manuscript seeks to offer insights for researchers, funders, and decision-makers interested in innovative approaches for organizing and implementing coalitions dedicated to promoting child health and well-being.

### Child health equity coalitions

Community coalitions are coordinated groups that work together to promote the health of their communities by identifying health concerns and planning and implementing strategies to address them (12). According to a recent systematic review of coalitions, coalitions are defined by a focus on three types of coordination: knowledge coordination, negotiated coordination, and action coordination (13). Coalitions with a child health equity focus, otherwise known as child health coalitions, have become increasingly common in communities across North America. These coalitions work to address local health challenges related to food insecurity and healthy food access, healthy family meals, alcohol and drug abuse, mental health, and other concerns (14–19). While coalitions often bring organizational representatives together to collaborate on programs and community events, many are now focusing their work on influencing policies, systems, and environments to improve health outcomes (10, 20–22).

### Systems thinking, health equity, and transformative learning

**Figure 1:**
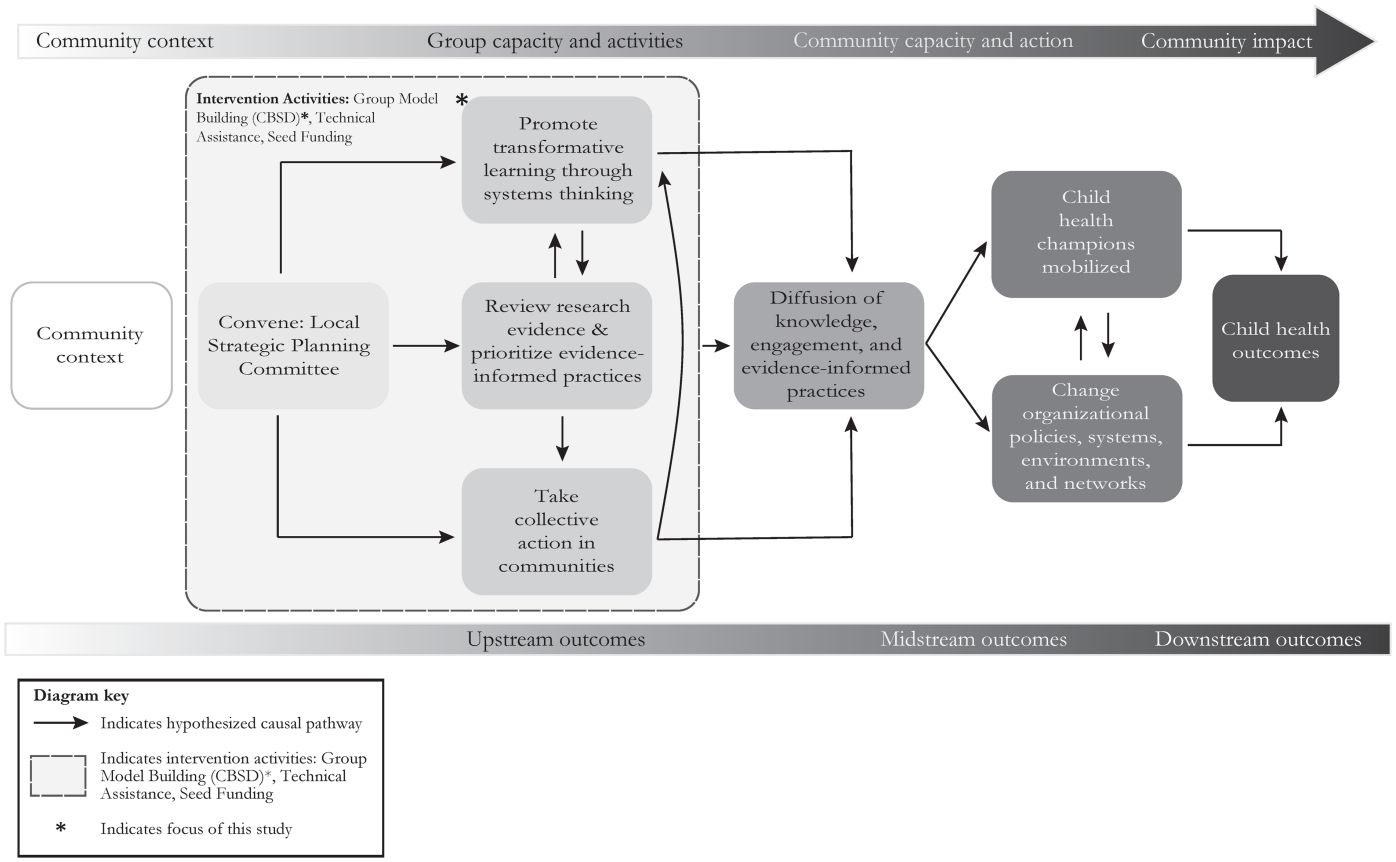
Thirteen members from the Milwaukee County Organizations Promoting Prevention coalition participated in Community-based System Dynamics activities as part of the Stakeholder-driven Community Diffusion (SDCD) theory-informed intervention. This intervention is a three-phase intervention to facilitate and evaluate a multisector group of stakeholders, review evidence, and set priorities for future work. In this study, our analysis of transformative learning included examining changes in systems thinking and health equity actions.

Visualized in Figure 1, our analysis of transformative learning included examining changes in systems thinking and health equity actions. Transformative learning, as described by Mezirow (23), is a process of profound personal and collective change in the way individuals perceive, interpret, and respond to their experiences and the world around them. This type of learning involves critical reflection, reassessment of assumptions, and the development of new perspectives or frames of reference (24). Measuring transformative learning requires assessing shifts in consciousness, self-awareness, and changed behavior (23, 25).

Systems thinking concept codes for our analysis were informed by Donella Meadows’ “Thinking in Systems” (26). Meadows defines systems thinking as a discipline for seeing wholes, a framework for seeing interrelationships rather than things, and for seeing patterns of change rather than static snapshots. It involves understanding elements such as feedback loops, stocks, and flows within systems and how these elements interact to produce the behavior of the system over time. Key components include recognizing interconnections, understanding complex cause-and-effect relationships, and identifying leverage points where interventions can create significant positive changes.

Concepts from health equity, specifically health equity actions, were adapted from Shiriki Kumanyika’s Getting to Equity framework (27), which translates the intention to achieve equity into action through four synergistic categories: “Increasing Healthy Options” (enhancing access to nutritious food), “ReCapacity” (mitigating factors discouraging healthier choices), “Improving Social and Economic Resources” (leveraging programs to address hunger, poverty, and disparities), and “Building on Community Capacity” (promoting community-driven policy, system, and environmental changes). In this study, the framework was applied post hoc to categorize participants’ thinking and actions to promote child health equity.

### Community-based System Dynamics and the Stakeholder-driven Community Diffusion theory-informed intervention

Thirteen members of the Milwaukee County Organizations Promoting Prevention coalition participated in Community-based System Dynamics (CBSD) activities as part of the Stakeholder-driven Community Diffusion (SDCD) intervention (Figure 1) (8). CBSD combines system dynamics with community engagement to help coalitions collaboratively analyze and address complex issues like health disparities (28, 29). By mapping out problem dynamics, anticipating intervention outcomes, and identifying leverage points, CBSD enables coalitions to assess the impact of their strategies and make more informed decisions.

The CBSD activities are part of the SDCD intervention, a three-phase process designed to facilitate and evaluate a multisector group of stakeholders, review evidence, and set priorities. The SDCD theory suggests that a coalition’s understanding and commitment to promoting healthy child weight will spread via social networks (30), leading to evidence-based actions that improve child health outcomes through policy, systems, and environmental changes (31). Specific intervention components (8, 22) and the underlying theories (32, 33) are detailed elsewhere.

The SDCD intervention is informed by the Community Coalition Action Theory, the Community-based Participatory Research model, and systems thinking (26, 34, 35). These frameworks explain how coalitions function and how participatory research can drive community-level change by building capacity and pooling resources (34–36). Systems thinking, which underpins the SDCD theory and activities, is also applied to modify, measure, and model changes in coalition members’ knowledge and engagement (9, 26, 37). Researchers advocate for using systems thinking and related methods, such as social network analysis, system dynamics modeling, and agent-based modeling, to address child health issues (38, 39).

### Community background

Milwaukee is Wisconsin’s most populous and diverse city, with a Diversity Index (DI) of 66.8%, compared to the state average of 37% and the US average of 61% (40). Seen in Table 1, Milwaukee County has higher poverty rates, lower employment, and lower home ownership than the rest of Wisconsin. It is also one of the most segregated areas in the US (41), with Milwaukee tied with Detroit as the most segregated city for Black residents and among the top five cities with the highest dissimilarity levels, showing little change over three decades (42).

**Table 1:** Summary of Milwaukee County and Wisconsin State demographics.

Community	Milwaukee County	Wisconsin State
Community characteristics (2021)^*^		
Population estimate	939,489	5,893,718
Land area (mi^2^)	241.5	54,153.1
Median household income (USD)	56,347	67,125
Bachelor’s degree or higher (%)	34.5	32.5
Foreign born (%)	9.2	5.1
Diversity index (%)	66.8	37
Employment rate (%)	61.2	62.7
Poverty	24.1	10.8
Population without health insurance coverage	10.3	5.4
Homeownership rate	40.9	68.1
Race and ethnicity (%)		
Hispanic or Latino (all races)	19.9	7.5
NH White	39.9	86.6
NH Black or African American	39.4	6.8
NH American Indian and Alaska Native	0.6	1.2
NH Asian	4.5	3.2
NH Native Hawaiian and other Pacific Islander	0	0.1
NH some other race	0.1	0.1
NH two or more races	7.6	2.2

* U.S. Census Bureau (40). American Community Survey 1-Year Estimates.

### The Coalition: Milwaukee County Organizations Promoting Prevention

The Milwaukee County Organizations Promoting Prevention (referred to as the “Coalition”) has been engaged in community and participatory research for 18 years, focusing on supporting a healthy community by promoting child, family, and residents health and well being. Their vision is to improve health outcomes through equitable partnerships and shared resources; offer ongoing streamlined education opportunities for members and target audiences; implement and support health promotion projects; engage in advocacy; and share information via consistent communication and networking opportunities (43). The Coalition has successfully developed four policies adopted by eight neighborhood centers, focusing on land use, professional development, healthy food and beverages, and active living (19, 43). Their approach is guided by an asset-based community development (ABCD) lens, which emphasizes building healthy outcomes to drive positive social change rather than merely reducing negative health consequences (44, 45). The ABCD framework has proven to be a strong motivator in community development initiatives (2, 46, 47).

## Methods

This case study uses an in-depth, contextual analysis to explore the perspective shifts and transformative learning that occurred during the CBSD activities. The research aims to understand how coalition members’ perspectives evolved throughout the intervention and to assess the implications of these changes for the Coalition’s future work. Grounded in the SDCD theory-informed intervention, which emphasizes changing mental models to address child health inequities and drive policy, practice, and environmental changes, the study is guided by two primary research questions: “*How did the Coalition members’ perspectives change?*” and “*What did this change mean for the Coalition’s future work?*”

To address these questions, the study uses a mixed-methods approach, incorporating qualitative semi-structured interviews and quantitative web-based survey data collected from 10 matched committee members before and after the intervention. Additionally, a causal-loop diagram was developed to map and analyze the systemic factors influencing child health. The Tufts University Institutional Review Board reviewed and approved this research.

The research team leveraged existing partnerships in Wisconsin to connect with changemakers—community leaders—who, in turn, helped identify multisector coalition members for the intervention. While the changemakers guided research activities, they did not directly participate in the intervention. Individuals from both the changemakers and the coalition member groups formed a collaborative writing team with the research team to write and review the paper.

### Intervention activities and sample

Described in Table 2, 13 coalition members participated in the CBSD portion of the intervention that was delivered over approximately 6 months between December 2020 and May 2021. Over a series of eight meetings, the research team conducted group model building sessions, a CBSD activity, a participatory process where facilitators guide groups through sets of structured activities to develop visual models representing the system structures that drive a central problem of interest over time (9). Details about group model building activities, including freely available scripts for planning and facilitating group model building activities, are available elsewhere (9). The research team distributed surveys and conducted interviews before and after the meeting series.

**Table 2:** Summary of coalition member characteristics.

Coalition characteristics	Description
Coalition size (*n*)	13
Bachelor’s degree and above (%)	84.6
Female (%)	76.9
Race/Ethnicity	
Black or African American	69.2% (*n* = 9)
White	30.8 (*n* = 4)
Coalition’s child age impact focus	0–5 y
Coalition focus area(s)	Promoting healthy weights in young children; Children eating healthy meals; Improve health status of children 0–5 by increasing resource coordination across the community; Advocacy for healthy environments

The initial meeting involved introductions and a discussion on the SDCD theory-informed intervention, ending with a “hopes and fears” activity (48). The second meeting introduced CBSD group model building, leading to consensus on two reference modes and discussions on causal loop diagrams. By the third meeting, smaller groups developed their diagrams. Between the third and fourth, the research team combined diagrams, presenting a unified causal loop diagram in the fourth meeting. The final synthesized diagram is reported on in the results section, used to triangulate themes across the pre/post web-based surveys and interviews. The fifth and sixth meetings involved reviewing evidence and prioritizing intervention topics. In the seventh and eighth meetings, the team supported community action, forming three working groups aligned with priority areas. Finally, the group convened once more in August 2021 to celebrate successes and review SDCD preliminary data.

### Data collection and instruments

Participants completed two data collection instruments, a web-based survey and an interview (described in Table 3), before the intervention and 6 months into the intervention, which aligns with when the CBSD activities are completed (Table 4).

**Table 3:** Semi-structured interview and web-based survey questions.

Pre-intervention question items	Scale
*Semi-structured interviews*	
1. What are your primary concerns regarding childhood healthy weights in Milwaukee?	Open
2. In your opinion, what are two of the main causes of unhealthy child weights in Milwaukee?	Open
3. In your own words, can you describe the relationship between child weight and healthy food access?	Open
4. In your opinion, what are the top two actions that should be prioritized in Milwaukee with respect to promoting healthy child weights?	Open
5. What do you view as the barriers to addressing child weight in Milwaukee?	Open
6. Please rank your perception of how efforts related to child healthy weights are prioritized within your organization.	Not a priority; somewhat of a priority; a major priority
7. Please rank your perception of how much your organization considers child weight to be a problem in Milwaukee?	Not a problem; somewhat of a problem; a major problem
8. How has your organization influenced community awareness, policies, and/or regulations related to promoting healthy child weight?	Open
*Web-based survey*	
1. Since joining the coalition, have you experienced a change in your perspective(s) related to *healthy weight for children in Milwaukee*?	If yes: open
2. Please describe what or who influenced this change.	Open
Post-intervention questions (asked in addition to the pre-intervention questions)	
*Semi-structured interviews*	
1. Are you exploring new roles, relationships, or actions on this topic within your work because of being involved with the coalition? Or if not, can you explain why that is?	Open
2. Has this exploration resulted in any course of action? If so, what? If not, why do you think that is?	Open
3. Briefly, can you reflect on the process we worked through with the coalition? What did you like and not like, and what is one thing you would suggest we change if we repeat this process in the future?	Open

**Table 4:** Data sources and data collection timeline.

	Intervention month						
	1	2	3	4	5	6	7+^*^
Interviews	X					X	
Web-based surveys	X					X	
Causal-loop diagram (developed/finalized)		X	X			X	
Research literature			X	X			X

* Ongoing intervention activities outside the scope of this study.

#### Semi-structured interviews

Two research members conducted semi-structured in-depth interviews with 12 coalition members pre-intervention and 10 coalition members post-intervention, for a total of 10 pre-post matched coalition member interviews. Interviews ranged from 30 min to an hour long. Summarized in Table 3, participants were asked a series of questions related to their perceptions and beliefs concerning child healthy weights, their organization’s prioritization of childhood healthy weight efforts, and their experiences working with the research team to influence policy, system, and environmental changes in their local community. The initial set of questions focused on personal beliefs, including concerns about child healthy weights in their community, causes of unhealthy child weights, the relationship between healthy weight and healthy food access, prioritized actions for child health promotion, and perceived barriers to promoting child healthy weight in their community. Next, participants were asked to assess their organization’s prioritization of child healthy weight efforts, the organization’s perception of promoting healthy child weights in their community, and their organization’s influence on community awareness, policies, and regulations related to the promotion of child healthy weight. Finally, participants were given the opportunity to explore any changes in their roles, relationships, or actions because of their involvement, along with reflections on the intervention process and potential improvements for future iterations.

#### Web-based survey

The Coalition members filled out a web-based survey that asked about perspective changes and what and who influenced perspective changes. Questions included “Since joining the coalition, have you experienced a change in your perspective(s) related to healthy weight for children in Milwaukee?” and “Please describe what or who influenced this change.”

### Analysis and triangulation

Ten matched pre-post interviews and 10 pre-post open ended survey answers were transcribed using an online transcription service. The transcribed interviews and surveys were then coded using two different methods. In the first method, two members of the research team thematically coded the interviews and surveys and generated categories of codes. These categories were then tallied to examine the change in frequency of code occurrences between pre- and post-intervention. The second method followed a more traditional route of qualitative coding using Nvivo, a qualitative coding and analysis software. The research team began by iteratively developing a comprehensive codebook that would serve as the foundation for our qualitative analysis. As seen in Table 5, this codebook included a combination of inductive and deductive codes. Initially, we developed the codebook to focus on systems thinking concepts, systems insights, and health equity action (health equity thinking codes were created inductively). Systems thinking concept codes were developed combining concepts from Meadows’ Thinking in Systems (26) and reviews of complex systems and their features (37, 49). System insights codes were developed using Hovmand’s levels of system insights (9). These codes were thematically categorized into surface, mid, and deep system insights by following a similar schematic outlined in Arnold and Wade’s 2017 article, that maps system thinking skills along a spectrum of low maturity (e.g., individual approaches a system from only one perspective) to high maturity (e.g., actively explores multiple, non-obvious perspectives, some of which might conflict with the thinker’s view). The resulting system insights themes exist along a spectrum from surface level insights (e.g., acknowledgement of a system) to deep level insights (e.g., anticipating systemic implications). Finally, health equity action categories were adapted from Kumanyika’s Getting to Equity framework, which consists of four categories, including interventions to reduce deterrents to healthy behaviors, building on community capacity, improve social and economic resources, and increase healthy options (27).

**Table 5:** Selected codes, code definitions, and data excerpts.

Selected code name	Definition	Data excerpt
Describing the food environment	Participant describes the environments in which people select and/or consume foods	“I believe food choices are heavily influenced by the surroundings and what’s easily accessible.”
Describing diet	Participant describes the kinds of foods one consumes	“…they tend to consume a lot of pre-packaged and processed foods because they are easy and quick.”
Describing diffusion concepts	Participant describes the spread of ideas, influence, engagement, or other factors related to promoting healthy child weight	“I’ve noticed a growing interest in promoting healthy child weight in our community.”
Describing complexity concepts	Participant recognizes that there are many people, organizations, values, interests, etc. influencing each other and influencing child weight trends broadly	“I see that child weight trends are influenced by numerous factors. It’s not just about personal choices.”
Acknowledgement of system components	Participant acknowledges that a system with components does exist but does not explain how its parts are interrelated	“I understand that there is a larger system that has to do with child weight, involving schools, families, media, and community resources.”
Identifying how system components are interrelated	Participant describes reciprocal relationships or interactions between the components, where the output or behavior of one component influences the input or behavior of another component, and vice versa.	“…the accessibility of healthy food in local stores seems to affect the eating habits of families, and those habits shape the demand for healthy food in stores…”
Connecting systems to emergent behavior	Participant describes what system structures influence the behavior of a specific system (e.g., identifying a reinforcing loop that has a large influence on system behavior at various points over time)	“…families have limited access to healthy food. Over time, this not only affects their health but also influences what food suppliers or stores offer…making healthier options even less accessible.”
Discussing social determinants of health	Participants describe conditions in which people are born, grow, live, work, and age that shape their health outcomes	“…a child’s environment has a significant impact on their health outcomes, especially when it comes to weight… like the safety of a neighborhood, the quality of schools…”
Discussing targeted interventions	Participant discusses implementing targeted interventions to address the specific needs and challenges faced by marginalized and underserved populations	“…design interventions specifically tailored to address the unique challenges faced by marginalized communities…”

After importing transcribed interviews in NVivo, and to ensure intercoder reliability, a member of the research team led a training session or “test” based on coding of a single interview. This was done by selecting the codes to be included in the “test,” the selection of previously coded/rated excerpts to comprise the test, and then specifying a name and description for the test. A member of the research team decided to include all deductive codes from the codebook to include for two research assistants to use for coding a single interview and survey. The two research assistants were prompted to access the test and to apply all codes to the set of excerpts making up the test. During the test, research assistants were blind to each other’s work. Upon completion, NVivo reported a Cohen’s Kappa of 0.64 indicating acceptable intercoder agreement. As we proceeded, each interview was deductively coded twice by two research assistants using the codebook and reviewed by a member of the research team. As new codes emerged, they were added to the codebook for both coders to apply (and in some cases reapply) to the interviews and surveys. For analysis, research assistants, under the supervision of a member of the research team, reviewed the codes, emergent categories, and examined how the categories changed from pre- to post-intervention regarding systems thinking and transformative learning.

Open-ended surveys and interviews were conducted to capture both the breadth and depth of coalition members’ perspective shifts during the intervention. Seen in Table 6, thematic analysis was used across the interviews and surveys. Themes were generated by comparing codes and categories from the interviews and survey based on the following criteria: frequency of occurrence (more than three times), relevance to research questions, consistency in appearing across data sources, and overall uniqueness from other themes.

**Table 6:** Themes, definitions, data sources, and triangulation process.

Theme	Definition	Data source	Triangulation process
Drivers of healthy child weights	Reflects the multifaceted elements influencing the health and weight of children	Semi-structured interview, Web-based survey	Compare codes from open-ended interview and survey, looking for consensus; identify most frequent, salient codes and create themes; cross-verify themes with causal-loop diagram; reflect on and refine theme to ensure theme is based on frequency, relevance, consistency across data sources, and uniqueness
Systems thinking concepts	highlights the recognition and understanding of the interrelationships between various elements that influence child health	Semi-structured interview, Web-based survey	
Surface level system insight	Indicates a basic awareness of the existence of systems or structures that contribute to child health	Semi-structured interview, Web-based survey, literature	Integrate deductive codes informed by Hovmand (9) with inductive codes; compare codes from open-ended interview and survey, looking for consensus; identify most frequent, salient codes and create themes; cross-verify themes with causal-loop diagram and original literature reflect on and refine theme to ensure theme is based on frequency, relevance, consistency across data sources, and uniqueness
Mid level system insight	Participant went beyond recognizing the existence of systems and exhibited an awareness of the interrelatedness and interactions between system components	Semi-structured interview, Web-based survey, literature	
Deep level system insight	Participants showcased an in-depth comprehension of the complex interplay between system structures and behaviors over time	Semi-structured interview, Web-based survey, literature	
Health equity thinking	Reflections on health disparities and the underlying determinants influencing health outcomes	Semi-structured interview, Web-based survey, literature	Integrate deductive codes informed by Kumanyika (27) with inductive codes; compare codes from open-ended interview and survey, looking for consensus; identify most frequent, salient codes and create themes; cross-verify themes with causal-loop diagram and original literature reflect on and refine theme to ensure theme is based on frequency, relevance, consistency across data sources, and uniqueness
Health equity action	References to actions or strategies aimed at addressing health disparities	Semi-structured interview, Web-based survey, literature	

The integration of quantitative analysis and qualitative results involved coding and categorizing the open-ended survey responses to align with the thematic findings from the interviews. This process was guided by the research team and changemakers, who worked together to ensure that the themes were comprehensive and accurately reflected participants’ experiences. To further validate these themes, they were cross-referenced with the causal-loop diagram factors generated during the CBSD meetings. The cross-validation involved broadly mapping the identified themes onto specific loops and variables within the CLD, assessing how well the themes aligned with the systemic dynamics represented in the diagram. For example, when a theme highlighted a systemic barrier or leverage point, it was examined against the feedback loops in the CLD to determine whether the qualitative data supported or expanded upon these relationships.

The entire research project, including changemakers, coalition members, and the research team, reviewed these integrated results during the writing process. This collaborative review allowed for further refinement and ensured that the final analysis accurately reflected the shifts in perspectives that occurred throughout the intervention. The themes were ultimately used to describe participants’ experiences from the initial meetings to the later formation of working groups focused on identified priority areas.

## Results

The following sections present the results from interviews and surveys, organized into three key themes that reflect the overall progression of the intervention. These themes are arranged chronologically to represent the stages of the intervention, from the initial convening of stakeholders to the implementation of local actions. The first theme, “Building Trust and Relationships for Systems Work,” captures the early stages of the intervention when committee members were brought together. The second theme, “Rethinking What Influences Healthy and Fit Kids,” emerges in the middle of the intervention as committee members collaborated to identify priority actions. The final theme, “Increasing Engagement and Sustaining Partnerships for Action,” represents the conclusion of the intervention, where committee members committed to taking local actions.

These themes illustrate the process of connecting stakeholders from different sectors who are concerned with child health in Milwaukee, WI. This process involved rethinking the various factors that influence child health and creating strong partnerships to promote and implement collective action. The diverse perspectives captured in these themes not only highlight the specific context of Milwaukee but also offer insights that may be relevant to other coalitions across the U.S. and to funders making decisions about community health priorities.

### Building trust and fostering collaborative systems thinking

When the Coalition formed, committee members knew from the outset that they “did not want this to be another public health thing” lacking ownership and perceived value to the broader community. They knew they were experienced in their respective areas but were looking for “a way to bring everyone together to work on promoting and enhancing children’s health.” They also espoused similar approaches to promoting child health, noting that collaboration, community voice, and empowerment were essential to realizing and sustaining impact. Through CBSD activities that helped express hopes and fears for the project, the Coalition expressed hope to make new partnerships across professional sectors and levels of service, as well as hope that the program would be ongoing and sustained. The Coalition expressed fear of losing momentum, of local politics, of not finding funding for future work, of not generating resident buy-in, and of not improving the health of hard-to-reach youth.

At the beginning of the SDCD theory-informed intervention, the Coalition varied in their perspectives on the main issues that needed to be addressed to promote child healthy weight in Milwaukee. A majority of members mentioned that children eating healthy meals, which included access to fresh, affordable, nutritious food, was the main issue that needed to be addressed. Less frequent were concerns around poverty, affordable housing, and transportation. Perspectives on the drivers or causes of child unhealthy weight were similarly varied. A majority of coalition members noted that little-to-no access to fresh, affordable, nutritious food to ensure children were eating healthy meals was the primary cause of children’s unhealthy weight. Other prominent causes mentioned were family habits and their influence on child behavior, unsafe neighborhoods and parks, physical inactivity, and food deserts. When asked about the barriers to intervening in these drivers, committee members most often mentioned that promoting children’s healthy weight is too complex, requiring changes in the parent–child relationship and an increase in the frequency and quality of family nutrition education. Less often they mentioned organizational barriers such as lack of capacity (i.e., money, personnel, time) and lack of information exchange.

The Coalition members reported that they knew that the main issues were tied together, as evidenced by how they described each. Coalition members used systems thinking concepts such as relationships, diffusion, complexity, system, and delay, to describe how these issues, drivers, and barriers were interrelated. For example, several coalition members described the time delays involved in increasing access to fresh, nutritious, affordable food, noting that improving access would require new relationships to form between community organizations to improve resource exchange and coordination. Less often, committee members used systems thinking concepts to explore surface, mid, and deep level systems insights otherwise known as levels of increasingly complex understanding of why child health inequities develop and persist. For example, several committee members referred to information exchange among the committee and the community (i.e., diffusion) as a component of cross-sector collaboration, which falls into the surface level insight category.

Overall, members of the Coalition in pre-intervention interviews agreed that past efforts to address child healthy eating and active living practices were not working in Milwaukee and that without cross-sector collaboration and mind-set shifts, promoting child healthy weight would continue to be viewed as too difficult. By reengaging their commitment to cross-sector collaboration and agreeing to learn more about what drives child health in Milwaukee, the Coalition was able to center community building and empowerment strategies as their organizing principles. Moreover, they agreed to engage in difficult conversations about the local issues in Milwaukee that put children at a disadvantage based on their lived experiences and knowledge.

### Rethinking what influences healthy and fit kids

Through these meetings, committee members shifted their perspectives on promoting child healthy weight away from an emphasis on addressing access to fresh, affordable, nutritious food and toward an emphasis on the impact of structural drivers such as racism. As one committee member noted, “institutional and structural racism is what really creates unhealthy environments which disproportionately affects marginalized populations, which tend to be populations of color and populations with low socioeconomic status.”

**Figure 2:**
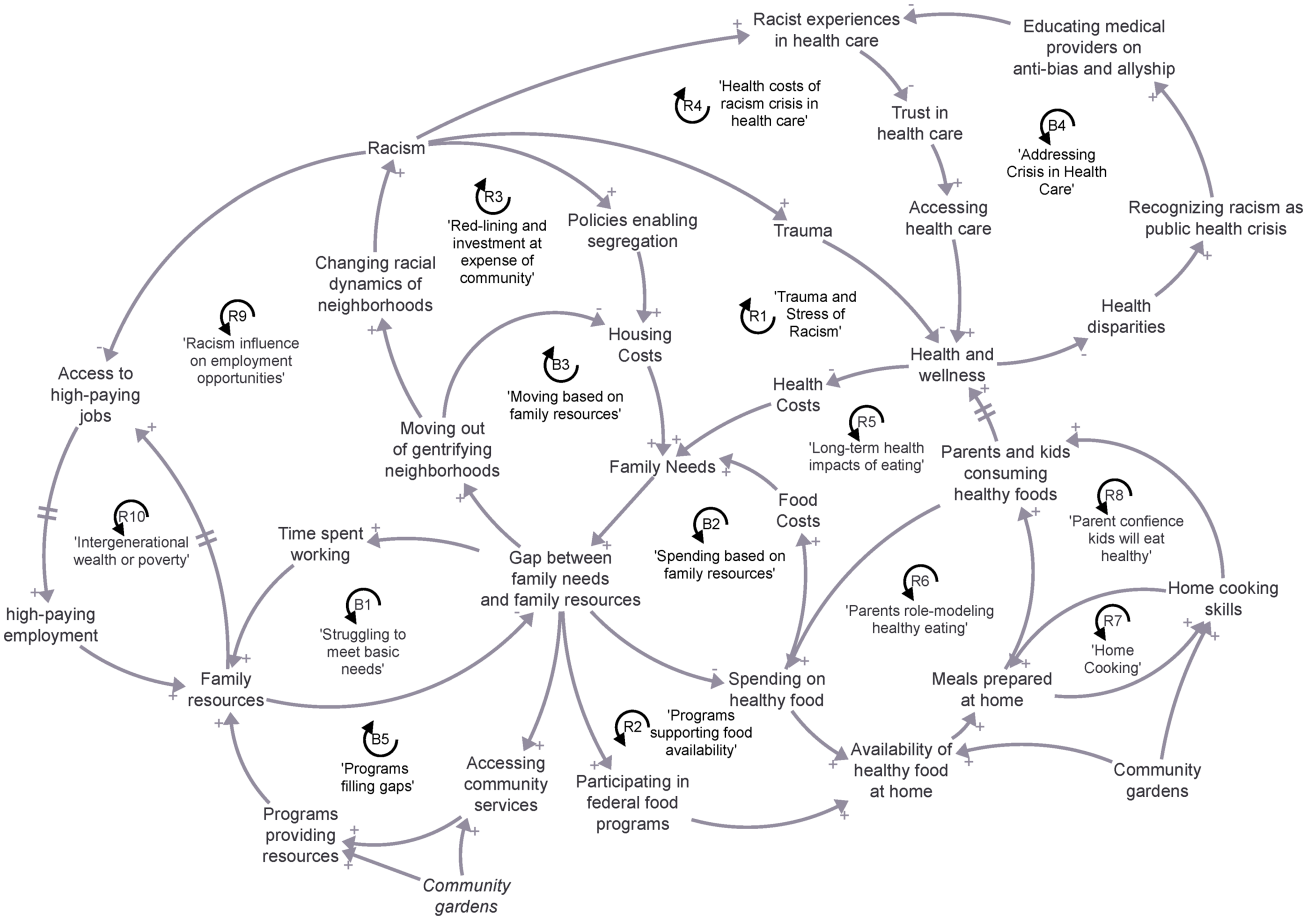
Causal loop diagram map of healthy eating, family resources, and the impact of racism in Milwaukee. Arrows with positive polarity indicate an increase in the causal variable leads to an increase in the receiving variable or a decrease in the causal variable leads to a decrease in the receiving variable. Arrows with a negative polarity indicate an increase in the causal variable leads to a decrease in the receiving variable or a decrease in the causal variable leads to an increase in the receiving variable. Reinforcing feedback loops (“R”) amplify changes over time. An increase leads to greater increases and decreases. Balancing feedback loops (“B”) dampen or limit changes over time. An increase feeds back around to a decrease, or a decrease feeds back to an increase.

The causal-loop diagram in Figure 2 depicts the Coalition members’ consensus view of the complex drivers of child healthy food consumption and healthy weight in Milwaukee. The diagram helps demonstrate the perspective shift that happened as the intervention unfolded, integrating initial perspectives about the food environment with a new emphasis on family resources and racism. The diagram illustrates several key features of child health in Milwaukee. Following different causal paths on the diagram, racism causes trauma that impacts health and wellness; racism directly influences trauma and “survival mode” stress, which can limit health and wellness practices (R1 Trauma and Stress of Racism). The diagram also depicts how families struggle to meet basic needs (B1 Struggling to meet basic needs). This has long term health impacts as families adjust their spending habits to try to close the gap between what they need and the resources they have. As seen in B2 (Spending based on family resources), when households are struggling to meet immediate needs, they do not spend as much on healthy food which creates additional health costs and burdens on the family in the long run. Limited resources sometimes mean that families do not have good options from which to choose. The gap between what families and individuals have and what they need means that some limited income residents with chronic diseases are making choices between buying healthy food and other necessities, such as paying rent or purchasing medication. R2 (Program supporting food availability) depicts accessing healthy food is enhanced with accessing food safety net benefits, helping to mitigate spending limitations. Affordability of healthy foods is influenced by income, ‘purchasing power’ and access to food safety net benefits (e.g., WIC, Food Share, Farmers Market Vouchers). Limited resources also influence where people live and shape neighborhoods (B3 Moving based on family resources). Some families that struggle to meet their needs are displaced from gentrifying neighborhoods to areas where housing costs are lower. This can create changing racial dynamics of neighborhoods that increase racism and policies enabling segregation and reinforce high housing costs and the struggle for families to meet resource needs (R3 red-lining and investment at expense of community). The diagram also explores how racism impacts trust in healthcare. As seen in R4, racist experiences in health care limit trust in providers causing people to access health care less, harming overall health and wellness. This leads to health disparities that have contributed to the recognition of racism as a public health crisis.

Post-intervention interviews provided additional context for the loops that appeared in the causal-loop diagram, revealing that coalition member concerns regarding promoting child healthy weight shifted to discussions around education, health disparities, racism, affordable housing, and social determinants of health such as socioeconomic status. Their perspectives on the main causes of unhealthy environments shifted to include more systemic factors like racism, affordable housing, and family habits and its influence on child health. When asked about the relationship between promoting child healthy weight and healthy food access, committee members shifted away from discussions about family habits and its influence on child health toward discussing socioeconomic status and public transportation. When discussing actions that should be prioritized to promote child healthy weight, committee members primarily discussed nutrition advocacy, nutrition education, and healthy cooking skill building for new mothers, families, and men. Though mentioned less frequently, addressing systemic racism, expanding federal programming and federal funding, and information exchange among child health organizations were new topics of conversation.

**Figure 3:**
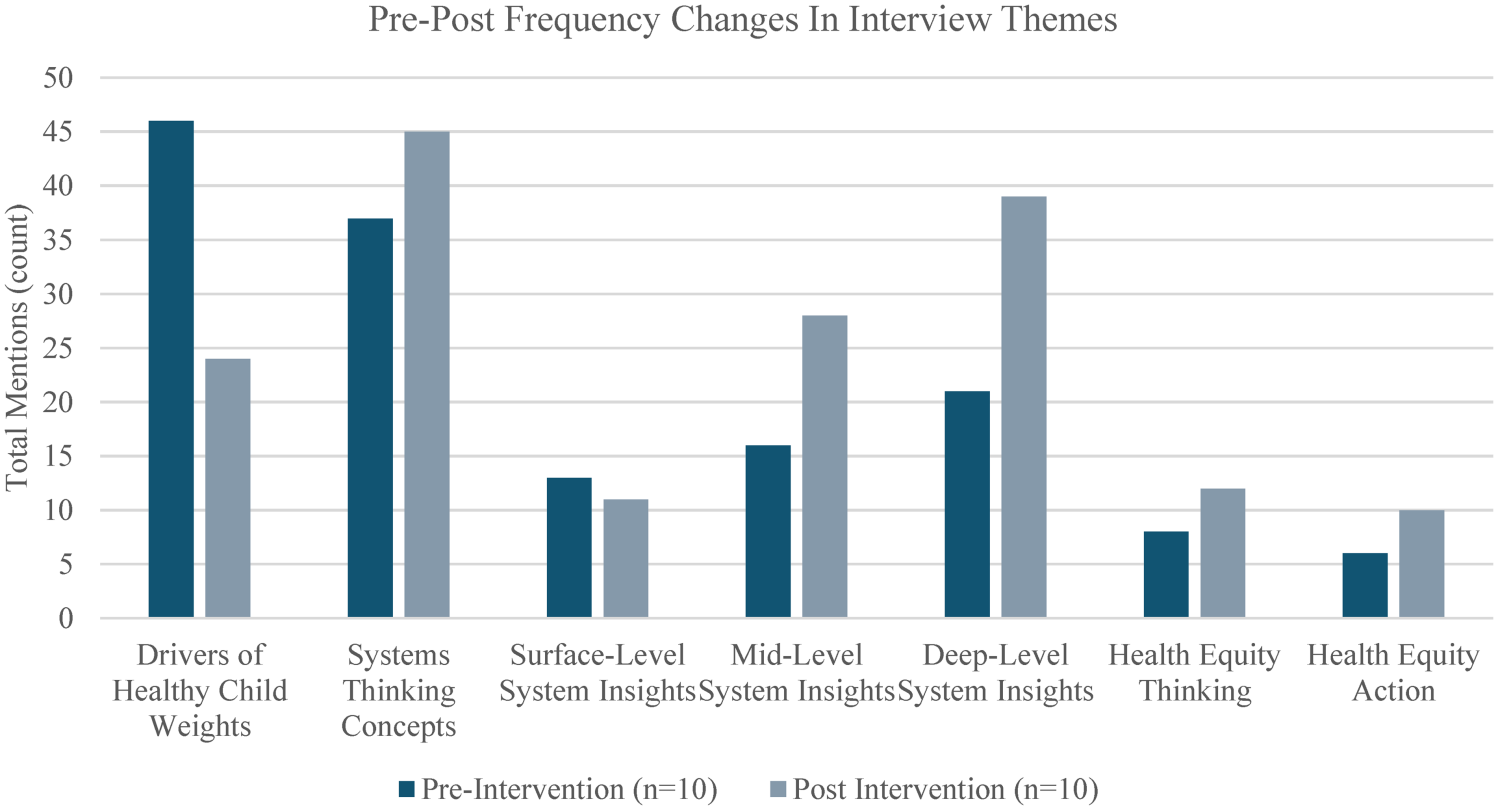
Pre-post frequency changes in codes that inform each theme.

Committee members also reported shifting their perspectives toward systems thinking and local action. As seen in Figure 3, which illustrates pre-to-post interview code frequencies, there were changes in what and how stakeholders were thinking about child healthy weight. In the pre-interviews, stakeholders primarily mentioned the food environment, policies, and cultural factors. They were also thinking about child healthy weight using systems thinking concepts such as collaboration, diffusion, and complexity. In the post interviews, stakeholders increased their systems thinking on topics related to child healthy weight and child health equity. Stakeholders made a large shift away from *what* drives child healthy weight and toward a broader category of *how* child health inequities are created and perpetuated, as evidenced by an increase in their deep system insights indicated by themes such as “why do things happen” and “what are the leverage points within the system.”

### Increasing engagement and sustaining partnerships for action

Despite initial fears about how the committee would unfold, several members of the Coalition noted in their post-intervention interviews how “people [lit] up” in discussions about promoting child healthy weight and how this shared energy brought momentum to the group to take action. Coalition members reaffirmed the need for sustaining the cross-sector collaboration created during these sessions. They noted that the success of cross-sector collaboration was the ability to hear diverse viewpoints, work with other committee members, discuss personal views, reflect on past committee work, and a space for concentrated thought about child health promotion. Finally, corresponding with the health equity action category from interviews, stakeholders increased their discussions about local change, thinking more about “next steps,” communicating these steps with others (diffusion), and how to apply their learnings to improve local conditions for children.

Coinciding with these perspective shifts toward sustaining partnerships and toward local action, coalition members worked to develop and sustain priority action areas based on the causal-loop diagram and the Coalition’s continued collaboration. Three working groups developed: (1) grants and sustainability; (2) advocacy and partnerships; and (3) community engagement and outreach initiatives. These three workgroups provided a holistic approach for empowering families to make healthier choices for themselves and their young children. The working groups also identified actions that could impact the system, some of which included: (1) advocating for policy or systems changes to promote racial equity, including needed neighborhood investments, housing supports for residents with low-incomes, educational and advanced learning opportunities, employment access, and living wages; (2) promoting nutritious eating patterns that support positive health outcomes across the life course, including breastfeeding for optimal growth and development in young children, eating to maintain a healthy weight at each life stage, and healthy aging; (3) educating medical providers on anti-bias and allyship to reduce racist experiences in health care, which can lead to improved health and wellness practices; and (4) supporting Black and Brown-owned businesses that offer healthy food to increase healthy food options and bring income into communities of color (50).

## Discussion and conclusions

This case study outlined a multi-phase intervention focused on promoting child health, answering the key research questions, “*How did the coalition members’ perspectives change?*” and “*What did this change mean for the coalition’s future work?*” The Coalition’s focus broadened from food access to discussions about racism, health disparities, affordable housing, and social determinants of health. The transition from addressing symptoms to delving into the root causes of health inequities showcased a heightened level of systems thinking and a readiness for local collective action. This transition can be seen in the Coalition’s causal-loop diagram, that demonstrates the integration of initial perspectives on food environments with broader influences such as family resource needs and racism. The diagram illustrates the complex factors affecting child health in Milwaukee, pointing to systemic issues that contribute to health disparities, including socioeconomic influences. Additionally, the Coalition committed to identifying priority collective action areas, emphasizing a more holistic approach toward improving child health. This evolution process can heighten the readiness of coalitions to address broader systemic issues, representing a significant step toward comprehensive community-informed interventions.

### Perspective shifts and systems thinking in Milwaukee

The shift in learning and perspectives that occurred among coalition members mirrors the broader shift in the field that integrates individual-level health outcomes and social and structural drivers of health. The Coalition began the intervention conceptualizing healthy food access, family habit influence on child eating behavior, and physical activity as the primary drivers of child healthy weight. They were also focused on building trust and relationships across sectors with those also interested in child health promotion. By the end of the intervention, the Coalition was focusing on more systemic drivers, such as structural racism and affordable housing, and continued to grow and build upon their trusting relationships to create collective action steps for transformative local change.

Several coalition members noted that the CBSD activities helped them visualize and name what was happening in the Milwaukee food environment, allowing them to integrate their perspectives and prioritize specific actions to promote child health weight. The process of perspective integration and priority action setting is important in complex food environments. As seen in Figure 2, the Milwaukee food environment has been shaped by redlining—and intentional blockbusting practices that promote quick housing sales resulting in “white flight”—with limited access to healthy food resources. Additionally, many Milwaukee residents live in areas classified as “food deserts” by the USDA, which indicates that those areas are both low-income and are a significant distance from a supermarket (51). Experiences of red-lining and food deserts can be integrated into diagrams, like the one in this study, that help contextualize the drivers and impacts of these experiences. This is especially important when considering health outcomes of these experiences, as low access to nutritious food is associated with chronic diseases like obesity, diabetes, hypertension, and some types of cancer (52). Importantly, by the end of the intervention, the process of perspective integration and priority setting was also marked by members considering *how* the drivers were connected. For example, while some committee members named red-lining as a factor that impacted child healthy weight in the beginning of the intervention, by the end of the intervention committee members were describing how red-lining was connected to racism, housing costs, and family resource needs. In their description, they also used more systems language such as “interrelationships” and “emergence.”

### Impact of the intervention on the Coalition’s future work

After engaging in CBSD activities together, the Coalition created three working groups: *Centering Health Promotion Using Community Gardens; Holistic Health and Wellness of Young Families, Pregnant Women, and Men*; and *Exploring Community Resources and Clinical Linkage Platforms.* The working groups led several community-based events between March–August 2021. Activities included conducting a “Community Garden Crawl” event at three community partner locations, with a variety of interactive health promotion activities for both parents and children; and co-hosting with Birthworkers United, Incorporated, the “It Takes a Village: Community Baby Shower and Resource Fair,” bringing together over 30 health and wellness vendors, a healthy “men who cook” chef’s grilling challenge, stress relieving and pampering service for expecting persons, kids healthy cooking and painting activities; haircuts for men and boys, baby clothing and supplies giveaways and raffles, and so much more. Positive participant feedback from both events identified that individuals wanted more skill building around gardening and healthy eating; that the stress relieving services and birthing resources were highly needed and appreciated; that they found their time was well spent and they learned a lot about self-care. The media coverage and donations from community baby drives mobilized the entire community to get involved. Regarding improving community resource coordination and understanding community-clinical linkage platforms, the Coalition was able to identify and establish a connection with a new effort in Milwaukee utilizing NowPOW and Unite Us technology. NowPOW and United Us are examples of emerging community information exchange technology being used to link health systems and community-based resources for streamlined and user-friendly client referrals to needed services.

Finally, the Coalition used the outcomes of this research to secure four additional grants: a one-year *CDC 2111 Closing the Gap Social Determinants of Health Accelerator Plan Grant for Milwaukee* (CDC 2111 SDOH Grant, 2021-2022) in partnership with the Wisconsin Department of Health Services (DHS), Chronic Disease Prevention Program (CDPP); the Healthy & Fit MCOPP-2 Project, as one of nine partner cities in the three-year *Catalyzing Communities to Impact Child Health Equity Grant* through Tufts University (2021-2024); the CDC Innovative Cardiovascular Health Grant (CDC 23-0005 Grant, 2023-2028) and the *CDC Addressing Conditions to Improve Population Health* (CDC 23-0058 ACTion Grant, 2023-2026) both continuing the partnership with the DHS, CDPP, thus generating and sustaining new partnerships and collective action initiatives.

### Implications for public health

By uncovering a shift in perspectives among coalition members—from addressing symptoms to understanding systemic factors driving child health—the study reinforces the need for more holistic, comprehensive public health interventions. Traditional public health initiatives often target symptoms rather than the root causes of health disparities (37). The study’s insights suggest a pressing need to reorient health interventions to address broader systemic issues affecting child health, such as racism, socio-economic factors, and structural inequalities. Embracing this approach would mean moving away from isolated, singular interventions toward more comprehensive, whole-of-community strategies that target underlying systemic determinants (53).

Additionally, the emphasis on a systems thinking approach underlines the importance of interdependencies and interactions within a community’s health ecosystem. Public health initiatives need to adopt a systemic perspective to comprehend the complex web of factors influencing health outcomes (54). Participants often remarked on how focusing on system structure and system dynamics demands collaboration across various sectors, including healthcare, education, housing, social services, and policymaking, to effect long-term positive changes. Public health interventions that integrate systems thinking principles could yield more enduring and transformative outcomes, ensuring better health equity and improved overall well-being for communities (6, 21).

### Implications for the study of health equity-focused coalitions

The study findings have implications for the study of coalitions and their role in addressing health disparities. Specifically, the research underlines the necessity for coalitions to integrate symptom-driven solutions with acknowledging and addressing the underlying systemic issues contributing to child health disparities. This deeper understanding suggests that coalitions must broaden their focus beyond isolated interventions, forming partnerships and initiatives that tackle the systemic drivers of health disparities.

While frameworks for achieving equity in child healthy weights are emerging, such as in Shiriki Kumanyika’s “Getting to Equity” framework (27), how coalitions are expected to promote equity is less clear. The process of visually externalizing mental models of the current environment (as seen in Figure 2) into a systems map through the CBSD group model building process, for example, offers a way forward for coalitions seeking to realize health equity in their local community. This is because externalizing mental models helps community stakeholders conceptualize different parts of the system from their own perspective as well as identify points within the system for personal and collective action. The process outlined is not without its challenges. Community stakeholders have different ideas about how child health and wellbeing can be promoted in their community. However, Coalition leaders’ long-standing respect within the community along with the building of trusting relationships through frequent contact and meaningful conversations during the CBSD activities, helped challenge normative ideas about impacting child healthy weight as seen later in the intervention (6).

### Implications for policy

The use of the SDCD theory-informed intervention, which features CBSD elements, and the resulting changes in coalition member perspectives has important implications for policy in Milwaukee and potentially more broadly. The complex drivers involved in promoting child healthy weight can be a significant challenge for policy makers. For example, the interconnected dynamics between housing discrimination and nutrition access in Milwaukee may lead policy design to overlook aspects of these dynamics that can be leveraged to promote healthy child weight. Thus, policy-making that engages the diverse group of local community stakeholders, incorporating their perspectives of these interconnected dynamics and their prioritized actions to promote healthy child weight, has a better chance of creating sustainable systems change. The causal-loop diagram map produced by the Coalition in this project is one example of capturing the diverse group of multi-sector community stakeholders’ perspectives, experiences, and prioritized actions to promote healthy child weight that policymakers can leverage in designing policies aimed at positive systems change.

Recognizing the importance of socio-economic resources and their significant impact on health disparities, the study also highlights that improving health equity requires comprehensive policy changes and societal shifts. Emphasizing long-term approaches in addition to immediate interventions is vital. By incorporating the study’s understanding of these determinants, coalitions can focus on systemic and structural change through advocacy, policy adjustments, and community outreach, thus fostering more meaningful and sustainable change that enables communities to thrive.

## Data Availability

The raw data supporting the conclusions of this article will be made available by the authors, without undue reservation.
